# Co-infectious Uveitis With Syphilis and Lyme Disease: A Case Report

**DOI:** 10.7759/cureus.58608

**Published:** 2024-04-19

**Authors:** Shobha Mandal, Hamnah Tayyab, Subhash C Mandal, Abdelhaleem Sideeg

**Affiliations:** 1 Internal Medicine, Guthrie Robert Packer Hospital, Sayre, USA; 2 Internal Medicine, B.P. Koirala Institute of Health Sciences, Dharan, NPL; 3 Hospital Medicine, Guthrie Corning Hospital, Corning, USA

**Keywords:** lyme disease, eye pain, blindness, ocular syphilis, uveitis

## Abstract

Uveitis is the inflammation of the uveal tract (i.e., iris, ciliary body, and choroid). Uveitis is categorized into the following three types based on the anatomical location of inflammation: anterior, intermediate, and posterior uveitis. Severe cases may lead to panuveitis, where all three layers may become inflamed potentially resulting in permanent vision loss. Uveitis can arise from different underlying disorders, including infectious causes or autoimmune disorders. Syphilis and Lyme disease are uncommon causes of uveitis. Eye involvement can occur at any stage in Lyme disease, characterized by diverse manifestations such as conjunctivitis, episcleritis, keratitis, uveitis, neuroretinitis, and retinal vasculitis. Patients may present with symptoms of blurred vision, eye pain or discomfort, visual floaters, headache, or intolerance to light. Patients can risk vision loss if not diagnosed and treated promptly.

## Introduction

Lyme disease is commonly caused by *Borrelia burgdorferi*, a spirochete transmitted by Ixodes ticks [1]. The disease affects multiple organs such as the skin, joints, heart, and nervous system [2]. The disease typically occurs in three stages if left untreated. Early stages classically consist of skin rash (erythema chronicus migrans, typically “bulls-eye” configuration is pathognomonic but not always present) associated with flu-like symptoms. Eye involvement can occur at any stage of the disease [1,2]. Ocular involvement of Lyme disease is characterized by diverse manifestations such as conjunctivitis, episcleritis, keratitis, uveitis, neuroretinitis, and retinal vasculitis. Patients with Lyme disease with severe ocular involvement are treated with tetracycline or beta-lactam antibiotics with/without steroids [1-3]. Syphilis is a sexually transmitted disease caused by *Treponema pallidum*, a spirochete. It can affect different parts of the eye, with the most common manifestation being uveitis. It is a rare condition that accounts for only 1-2% of all uveitis cases. Other presentations include conjunctivitis, episcleritis, retinitis, keratitis, iridocyclitis vitritis, chorioretinitis vasculitis, and papillitis. Syphilis is treated with penicillin antibiotics depending on the stages of syphilis, i.e., primary, secondary, and tertiary [4,5].

## Case presentation

**Figure 1:**
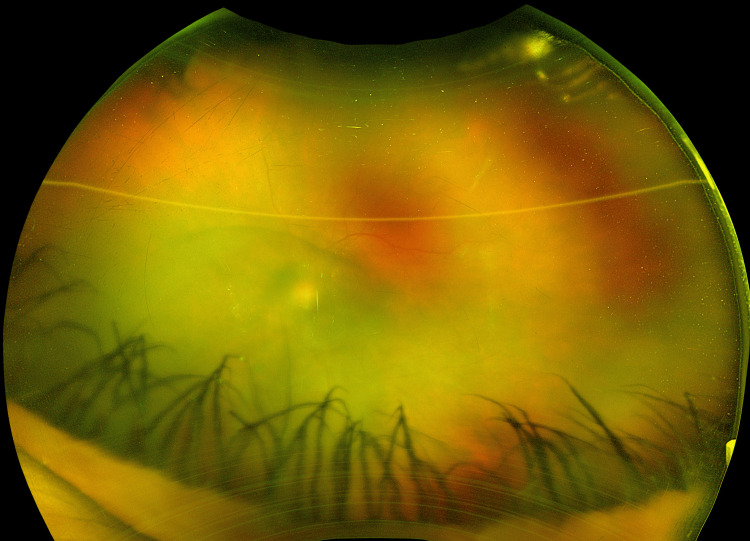
Left eye: 2+ anterior chamber cells and 1+ flare with posterior synechiae of the iris.

A 45-year-old female with a past medical history of hypertension presented to the emergency department with a complaint of severe bifrontal headache (9/10 intensity) associated with left eye pain and blurring of vision. She also had rashes on her palms, soles, and torso which started a few weeks back. Initially, she tried over-the-counter pain medications without any improvement. Her genitals were normal, and there was no history of known tick bites. The patient came to the emergency department for further evaluation because of the worsening symptoms. She had developed blurring of vision in the right eye with floaters and oozing of clear fluid from both eyes. Physical examination including neurological examination was normal. Extraocular muscle testing indicated full movement in both eyes. An ophthalmology consultation revealed visual acuity with/without correction near the right eye as 20/40 and in the left eye as very low, with only perception of hand movement. Pharmacologically dilated pupils showed 360-degree posterior synechiae in the right eye and a secluded pupil in the left eye. Intraocular pressure measured with a Tonopen was 9 mmHg in the right eye and 8 mmHg in the left eye. Dilation with atropine in the right eye revealed a hazy vitreous with the presence of trace cells, the optic disc appeared sharp and pink, the macula was normal, blood vessels exhibited a normal course and caliber, and the periphery displayed semi-transparent outer retinal whitening inferonasally with multifocal deep yellow opacifications. The left eye was not visualized upon atropine dilation. The left eye had 2+ cells in the anterior chamber cells and 1+ flare with posterior synechiae of the iris (Figure 1).

The diagnosis of panuveitis without macular edema or vasculitis was made after ophthalmology consultation and she was started on steroid and atropine eye drops without much improvement. A few days later, she developed a fever, sore throat, and generalized lymphadenopathy. She had a history of intravenous drug use and needle sharing with her boyfriend with whom she was involved in regular unprotected sex for a few years. She reported having hepatitis C with the last treatment received one year back. Laboratory workups revealed abnormal rapid plasma regain (RPR titer 1:512 high) (Table 1) and Lyme serology (Lyme disease antibody screen index: 1.37; high: <0.90, negative: 0.90-1.09, equivocal: >1.09 positive) (Table 2). Other serology and workups including human immunodeficiency virus (HIV)-1/HIV-2 antibody and HIV-1 P24 antigen were unremarkable for autoimmune and infectious.

**Table 1:** Syphilis serology showing reactive rapid plasma reagin and fluorescent treponemal antibody absorption.

Syphilis serology	Result
Rapid plasma reagin	Reactive
Fluorescent treponemal antibody absorption	Reactive

**Table 2:** Lyme serology showing reactive immunoglobulin G (IgG) and immunoglobulin M (IgM).

Lyme (*Borrelia burgdorferi*) serology	Result
18 kD IgG	Reactive
23 kD IgG	Non-reactive
23 kD IgM	Reactive
26 kD IgG	Reactive
30 kD IgG	Reactive
39 kD IgG	Reactive
39 kD IgM	Non-reactive
41 kD IgG	Reactive
41 kD IgM	Non-reactive
45 kD IgG	Non-reactive
58 kD IgG	Reactive
66 kD IgG	Non-reactive
93 kD IgG	Reactive
Ab IgG WB	Positive
Ab IgM WB	Negative

She was treated with intravenous penicillin (14-day course) and oral doxycycline for 21 days while continuing steroids and atropine eye drops. Symptoms gradually improved, and she was discharged with the peripherally inserted central line to complete an outpatient course of intravenous penicillin with the assistance of social workers. A recommendation for outpatient follow-up with her primary care provider in one week was made.

## Discussion

Uveitis is the inflammation of the middle layer of the eyeball, the uvea. Uveitis is of three types, namely, anterior uveitis, intermediate uveitis, and posterior uveitis. In severe cases, all three layers are inflamed, referred to as panuveitis, and may lead to permanent vision loss [6,7]. The causes of uveitis may be infections, autoimmune conditions, and sometimes trauma. Syphilis and Lyme disease are rare causes of uveitis and account for fewer than 1% of cases [7]. The seropositivity for Lyme disease can be incidental and IgM and IgG may persist for years without reactivation; hence, screening for borreliosis in all uveitis cases is not advised [2]. Posterior uveitis and panuveitis are more common than anterior uveitis in syphilis. Most patients with syphilis uveitis are co-infected with HIV which is associated with worse visual outcomes [8,9]. However, co-infection of syphilis and Lyme disease in patients with uveitis is very rare. To our knowledge, the patient described here appears to be the first documented case in the literature demonstrating concurrent co-infection of syphilis and Lyme disease. The patient did not have any typical symptoms related to Lyme disease or syphilis on the initial presentation. There was no known history of any tick bites.

As noted in numerous previous studies, progression to panuveitis can occur without typical signs and symptoms of syphilis and Lyme disease. In this case, the patient experienced progressive deterioration of vision, and the diagnosis of panuveitis was established after ophthalmology, as there was no satisfactory response to analgesic and topical steroids. To determine the underlying cause, a serological test for *Borrelia* and syphilis was conducted, revealing an incidental and unexpected finding of simultaneous co-infection. The broad application of this diagnostic testing is a subject of controversy as excessive testing of individuals with non-typical clinical symptoms and false-positive laboratory results lead to misdiagnosing many patients with Lyme-associated uveitis [10]. Although Lyme disease and syphilis screening is not routinely conducted for every case of uveitis, serology should be considered on a case-by-case basis when there is a high suspicion of these infections. Antibiotics and steroids are effective treatments in all uveitis patients. Ocular syphilis is treated similarly to neurosyphilis, with up to two weeks of intravenous penicillin supplemented with systemic corticosteroids that minimize eye inflammation and prevent any worsening from Jarisch-Herxheimer reaction [7]. Despite the preference for administering intravenous ceftriaxone in treating Lyme-uveitis, this patient responded well to three weeks of oral doxycycline. A retrospective study conducted by Bernard et al. between 2003 and 2016 revealed that the recurrence following treatment of Lyme-associated uveitis is common (four out of seven patients) and may require second antibiotics. Three hypotheses that can be accounted for the persistence and reappearance of symptoms are (1) reinfection, (2) relapse of original infection, and (3) autoimmune reaction [11].

## Conclusions

Concurrent co-infection of syphilis and Lyme disease is an unusual occurrence and can be associated with poor visual outcomes if treatment is delayed. These conditions can be easily missed as typical presentations are not always evident. Serology should be performed to detect these uncommon causes when suspicion is high. Because these diseases can be treated successfully, knowledge of their diagnosis and therapy is crucial. Timely referral to the ophthalmologist and treatment is vital in preventing blindness, as in our patient.

## References

[1] (2023). Lyme disease-associated uveitis: a case report and review emphasizing the importance of travel history and geographic considerations. https://morancore.utah.edu/section-09-intraocular-inflammation-and-uveitis/lyme-disease-associated-uveitis/..

[2] Issa R, DeSouza SA (2021). Recurrent bilateral chorioretinitis with positive Lyme serology: a case report. J Med Case Rep.

[3] Kılıç Müftüoğlu İ, Aydın Akova Y, Gür Güngör S (2016). A case of Lyme disease accompanied by uveitis and white dot syndrome. Turk J Ophthalmol.

[4] Pan SW, Yusof NS, Hitam WH, Noor RA, Embong Z (2010). Syphilitic uveitis: report of 3 cases. Int J Ophthalmol.

[5] Teixeira AM, Meireles E, Pereira Fontes C, Manuel M (2022). Ocular syphilis: a case report. Cureus.

[6] (2022). American Academy of Ophthalmology. What Is uveitis?. https://www.aao.org/eye-health/diseases/what-is-uveitis.

[7] Mustafa M, Muthusamy P, Hussain SS, Shimmi SC, Sein MM (2014). Uveitis: pathogenesis, clinical presentations and treatment. IOSR J Pharm.

[8] Bollemeijer JG, Wieringa WG, Missotten TO, Meenken I, ten Dam-van Loon NH, Rothova A, Los LI (2016). Clinical manifestations and outcome of syphilitic uveitis. Invest Ophthalmol Vis Sci.

[9] Amaratunge BC, Camuglia JE, Hall AJ (2010). Syphilitic uveitis: a review of clinical manifestations and treatment outcomes of syphilitic uveitis in human immunodeficiency virus-positive and negative patients. Clin Exp Ophthalmol.

[10] Rifkin LM, Vadboncoeur J, Minkus CC (2021). The utility of Lyme testing in the workup of ocular inflammation. Ocul Immunol Inflamm.

[11] Bernard A, Seve P, Abukhashabh A (2020). Lyme-associated uveitis: clinical spectrum and review of literature. Eur J Ophthalmol.

